# Cardiac myosin-binding protein C: a potential early biomarker of myocardial injury

**DOI:** 10.1007/s00395-015-0478-5

**Published:** 2015-04-03

**Authors:** James O. Baker, Raymond Tyther, Christoph Liebetrau, James Clark, Robert Howarth, Tiffany Patterson, Helge Möllmann, Holger Nef, Pierre Sicard, Balrik Kailey, Renuka Devaraj, Simon R. Redwood, Gudrun Kunst, Ekkehard Weber, Michael S. Marber

**Affiliations:** 1 King’s College London BHF Centre, The Rayne Institute, St Thomas’ Hospital, 4th Floor Lambeth Wing, Westminster Bridge Road, London, SE1 7EH UK; 2 Department of Cardiology, Kerckhoff Heart and Thorax Center, Bad Nauheim, Germany; 3 Department of Anaesthetics and Intensive Care, King’s College London BHF Centre, King’s College Hospital, London, UK; 4 Institute of Physiological Chemistry, Martin Luther University Halle-Wittenberg, Halle, Germany

**Keywords:** Ischemia, Myocardial infarction, Angina

## Abstract

Cardiac troponins are released and cleared slowly after myocardial injury, complicating the diagnosis of early, and recurrent, acute myocardial infarction. Cardiac myosin-binding protein C (cMyC) is a similarly cardiac-restricted protein that may have different release/clearance kinetics. Using novel antibodies raised against the cardiac-specific N-terminus of cMyC, we used confocal microscopy, immunoblotting and immunoassay to document its location and release. In rodents, we demonstrate rapid release of cMyC using in vitro and in vivo models of acute myocardial infarction. In patients, with ST elevation myocardial infarction (STEMI, *n* = 20), undergoing therapeutic ablation of septal hypertrophy (TASH, *n* = 20) or having coronary artery bypass surgery (CABG, *n* = 20), serum was collected prospectively and frequently. cMyC appears in the serum as full-length and fragmented protein. Compared to cTnT measured using a contemporary high-sensitivity commercial assay, cMyC peaks earlier (STEMI, 9.3 ± 3.1 vs 11.8 ± 3.4 h, *P* < 0.007; TASH, 9.7 ± 1.4 vs 21.6 ± 1.4 h, *P* < 0.0001), accumulates more rapidly (during first 4 h after TASH, 25.8 ± 1.9 vs 4.0 ± 0.4 ng/L/min, *P* < 0.0001) and disappears more rapidly (post-CABG, decay half-time 5.5 ± 0.8 vs 22 ± 5 h, *P* < 0.0001). Our results demonstrate that following defined myocardial injury, the rise and fall in the serum of cMyC is more rapid than that of cTnT. We speculate that these characteristics could enable earlier diagnosis of myocardial infarction and reinfarction in suspected non-STEMI, a population not included in this early translational study.

**Electronic supplementary material:**

The online version of this article (doi:10.1007/s00395-015-0478-5) contains supplementary material, which is available to authorized users.

## Introduction

Acute myocardial infarction (AMI) carries an increased risk of death and recurrent ischemia and therefore must be rapidly identified and differentiated from other causes of chest pain [7]. Cardiac necrosis biomarkers have become crucial in affirming or excluding AMI in suspected non-ST elevation acute coronary syndromes (NSTE-ACS) and are obligatory to make the diagnosis in an appropriate clinical context [31]. Cardiac troponins (cTn) have emerged as the gold standard biomarker and are incorporated in the universal definition of AMI [31]. However, the cTns have potential shortfalls and new necrosis biomarkers could prove invaluable [10].

Early generations of cTn assays lacked the sensitivity to detect the low concentrations that occur in the blood during the first few hours following symptom onset. This is because the cTn concentration does not peak until 16–18 h after chest pain onset [16, 25]. The latest generation of so-called “high-sensitivity (hs)”, cTn assays strive for reliable detection of low cTn concentrations in an attempt to diagnose and treat NSTE-ACS early [17, 26]. However, triage is confounded by the assays’ decreased specificity for AMI when used in this way since it is necessary to heed cTn concentrations close to the 99th percentile of a healthy population [8] and even then up to 25 % of patients with an eventual diagnosis of AMI are below this threshold at presentation [13]. Furthermore, although initial reports suggested that these assays allow more rapid diagnosis of AMI when the event is defined by a classic cTn assay [17, 26], this advantage is probably lost when contemporary high-sensitivity assays are also used to define the index event [12]. In view of these limitations in sensitivity and specificity, contemporary guidelines recommend repeat testing for diagnostic certainty. Despite a number of myocardial injury biomarkers being released more rapidly, none have surpassed cTn, principally due to their lack of comparable cardiac-selective expression. Consequently, current investigations focus on analyses of the magnitude of temporal changes in serum cTn concentration in order to improve the assays’ low positive predictive value for early diagnosis of AMI [27]. However, it is still unclear whether this strategy offers a clear advantage [1] and, if it does, what magnitudes of percentage or absolute change in concentration overcome the compounded noise of analytic and biologic variation [20]. Furthermore, it is likely that the magnitude of such change parameters (delta in concentration) are likely to be gender, age and vendor specific, further complicating their implementation [20]. Thus, the ideal marker would be analogous in temporal profile of release to cytosolic proteins such as creatine kinase, fatty acid-binding protein and myoglobin, but possess the cardiac-restricted expression, and thus organ-specificity and dynamic range, of cTns.

The sarcomeric protein, cardiac myosin-binding protein C (C-protein, MYBPC3, cMyBP-C or cMyC), is one such candidate we discovered following a systematic proteomic analysis of coronary effluent from ischemic mouse hearts [15]. cMyC is predominantly a thick filament-associated protein and is among the most abundant of cardiac proteins, ranked 19th out of over 2300 proteins quantified and is at least twice as abundant as cTnI and cTnT which are ranked 92nd and 118th, respectively [3]. cMyC has three distinct isoforms encoded by different genes: slow skeletal, fast skeletal and cardiac. The latter possesses a unique N-terminal domain as well as other specific features [28] that could serve as cardiac-restricted epitopes. We previously detected cMyC in mouse [15] and human [4] sera following myocardial infarction. Other investigators have subsequently also reported its elevation in patients with myocardial infarction [11, 21]. However, none of these previous cross-sectional studies have quantified the absolute magnitude or kinetics of release in comparison to cTn. Since the timing and amount of myocardial injury in NSTE-ACS is uncertain at presentation, our current study used other, less clinically relevant, models of myocardial injury to undertake detailed temporal comparisons.

## Materials and methods

### Animals

All experiments were performed in accordance with the United Kingdom Home Office Guidance on the Operation of Animals (Scientific Procedures) Act 1986, published by Her Majesty’s Stationary Office, London, UK and with the Guide for the Care and Use of Laboratory Animals published by the United States National Institutes of Health (NIH Publication No. 85-23, revised 1996).

### Isolation, culture and simulated ischemia (SI) of adult rat ventricular myocytes (ARVMs)

Ventricular myocytes were isolated from the hearts of adult male Wistar rats (200–500 g) by collagenase-based enzymatic digestion as described previously [5, 14]. Briefly, cells were pelleted and resuspended in warmed modified M199 culture medium (invitrogen) containing 2 mM creatine, 2 mM carnitine and 5 mM taurine. Adherence to laminin-coated plates over 2 h was observed, media changed and viable healthy ARVMs used for subsequent protocols. Cells were cultured on 6-well plates and washed once with phosphate-buffered saline (PBS) before the addition of 2 mL of either basic or ischemia buffer. Simulated ischemia comprised metabolic blockade (with 20 mM 2-deoxyglucose, 20 mM sodium lactate and 1 mM sodium dithionite) in a buffer lacking glucose, but containing high potassium [14, 24]. Ischemia was continued for up to 60 min with samples time-matched to controls in control buffer. Cells were then either prepared for confocal microscopy as described below, or for 1D SDS-PAGE and immunoblotting by fractionation into 2× SDS sample buffer containing 2-mercaptoethanol using a previously published protocol [9].

### Coronary effluent collection and analysis

Hearts were perfused on a Langendorff apparatus as described previously [15] with the following modifications. To limit non-ischemic damage to the heart and thereby non-specific protein release, an intraventricular balloon was omitted. Hearts were stabilized for 30 min after initiation of retrograde perfusion with a standard protein-free buffer (Krebs-Henseleit buffer) at a constant pressure to allow a complete washout of blood and limit the contamination of coronary effluent by plasma proteins. Fluorescently labeled polystyrene microspheres (15 µm FluoSpheres^®^, Invitrogen) were added to the side arm of the aortic cannula over 2 min to reduce the coronary flow to between 50 and 70 % of baseline. The coronary effluent was collected after 1, 2, 5, 10, 20, 30, 40 and 60 min in 2 mL aliquots into Triton X-100 at a final concentration of 0.05 % on ice to reduce the absorption of protein. Aliquots were immediately frozen in liquid nitrogen. Time-matched control hearts were identically perfused but not subjected to ischemia. Coronary effluents were thawed and kept at 4 °C at all times. Coronary effluent was concentrated using an Amicon-Ultra column (3 kDa cutoff, Millipore). Subsequent studies were carried out on the same volume of coronary effluent (1D gel) to reflect the physiology of acute myocardial infarction. Infarction was confirmed by visualization of microspheres in the heart on confocal microscopy and triphenyl tetrazolium chloride (TTC) staining of the hearts after fixation and sectioning as described previously [15].

### Left anterior descending coronary artery ligation in mice

Coronary artery occlusion was achieved using the hanging weight system [6]. Briefly, mice were anesthetized by isoflurane inhalation and ventilated by tracheal intubation. After left thoracotomy, hearts were subjected to 30 min of ischemia and varying times of reperfusion from 30 s to 2 h. Blood was collected by aortic bleed at the end of reperfusion to avoid myocardial protein contamination through direct cardiac puncture. Blood was kept at room temperature to allow clotting and then centrifuged at 10,000*g* for 15 min at 4 °C with serum aliquots subsequently stored at minus 80 °C. Hearts were then fixed for sectioning.

### Immunoblotting of rodent samples

Concentrated coronary effluents were separated by SDS-PAGE and immunoblotted using previously described protocols [15]. The ARVMs were not concentrated, but separated into fractions of cytosol, membrane and an insoluble fraction (myofilament) by a previously described method [30]. The following primary antibodies were used against troponin T (Abcam, catalogue no. 45932), troponin I (Cell Signaling Technology, catalogue no. 4002) and an affinity-purified rabbit polyclonal antibody directed against the N-terminal cardiac-specific region of cMyC (Prof. M. Gautel, King’s College London and [15]).

### Human serum sample collection, preparation and analysis

All participants gave informed consent before recruitment and all investigations conform to the principles of the Declaration of Helsinki.

In the STEMI cohort blood samples were obtained 2 hourly from presentation up to 18 h after symptom onset. One further sample was taken 24 h after symptom onset. Serum was taken from patients admitted to the coronary care unit at St Thomas’ hospital, London (UK) or the coronary care unit of the Dijon University hospital, Dijon (France). Since we knew our immunoblot technique was insensitive, analyses were restricted to patients with a cTnT rise of at least 2000 ng/L based on their blood draw 12 h after admission that was collected and analyzed as part of routine clinical care. The samples were kept at room temperature for 30 min to allow clotting and then spun at 4 °C before being aliquoted and frozen at −80 °C. For immunoblots, human serum was depleted of high-abundant protein by a multiple affinity removal system spin cartridge (Agilent, catalogue # 5188-8825). Serum was analyzed to ensure cMyC stability at room temperature and in storage and also to ensure equivalence between serum before and after protein depletion. The serum was concentrated prior to analysis and 150 µg of total serum protein was separated on a discontinuous 7 %/10 % acrylamide 1D SDS-PAGE gel, transferred to nitrocellulose and immunoblotted using proprietary polyclonal rabbit or monoclonal mouse antibodies raised against the cardiac-restricted C0C2 portion of cMyC (see below). Band density was measured by laser densitometry (GS800 scanner) and plotted against laboratory values of cardiac troponin T (Roche Elecsys^®^ platform) as a percentage of the maximal density/concentration at peak.

The TASH cohort and procedure for blood sampling has been described previously [22]. Samples from 20 patients undergoing this procedure were chosen solely on the basis of adequate serum volume and complete data sets. In 15 of these patients, high-sensitivity (hs) cTnT estimations were performed as previously described [22]. In five patients, hscTnT had not previously been measured and the analysis was performed on an Elecsys E170 (Roche Diagnostics).

The CABG cohort comprised patients in the control group of a completed randomized trial to examine the potential cardioprotective effects of ciclosporin using biomarkers of necrosis (Multicentre Research Ethical Committee (MREC), reference number 06/Q0502/83 and with the ISRCTN Register, reference number 49989273). Cardiopulmonary bypass was with a non-pulsatile flow, and a membrane oxygenator. Cardio-protection was provided by antegrade cold-blood cardioplegia or by cross-clamp fibrillation. The serum samples were chosen from 20 patients recruited from a single center and selected on the basis of available residual serum volume at each time point of collection. hscTnT analysis was performed on an Elecsys E170 (Roche Diagnostics). All cTnT assays were performed using contemporary reagent kits where the 99th percentile of a healthy reference population was reported at 14 ng/L with an imprecision corresponding to 10 % CV at 13 ng/L, limit of blank at 3 ng/L and limit of detection at 5 ng/L.

### Generation of anti-cMyC monoclonal antibodies

Fourteen 3-month-old Balb/c mice were immunized with either 20 µg of the recombinant C0C2 domains of cMyC or four overlapping peptides spanning the sequence of the C0 region or the recombinant C5 domain of cMyC dissolved in 200 µL of NaCl (0.154 M) and emulsified in 300 µL of complete Freund’s adjuvant (CFA). The peptides had an additional extension of five lysine residues at their N-termini. To increase the immunogenicity of these peptides, they were incubated with fructose for 2 h at 50 °C. Immunization was continued with another intraperitoneal injection of 20 µg of the antigens in NaCl (0.154 M) and 300 µL incomplete Freund´s adjuvant (IFA) 48 days after the first injection. 18 days later mice received a first booster injection of 20 µg antigen and a second booster 24 h before the hybridization on day 82 of the immunization process.

Mouse spleen cells (1 × 10^8^) and cells (1 × 10^8^) of the mouse myeloma cell line P3X63Ag8.653 were thoroughly mixed and cell fusion was mediated by polyethylene glycol 1500 (Boehringer Mannheim; Germany) [19]. Fused cells were cultured in RPMI-based HAT selection medium (100 µM hypoxanthine, 4 mM aminopterine, 160 µM thymidine) for 3 weeks and another 2 weeks in HT medium. Surviving hybridomas were grown in RPMI 1640 medium supplemented with 10 % fetal calf serum (SIGMA), HEPES (4.77 g/L), glucose (2.5 g/L), 2-mercaptoethanol (1 mM), l-glutamine (2 mM), insulin (5 mg/L) and gentamycin (80 mg/L).

### ELISA for the detection of mAbs to human cMyC

Costar high-binding immunoplates (96 wells) were incubated overnight with 50 µL of the recombinant C0C2 domain of human cMyC, the relevant peptides or a 28mer peptide of the C5 domain specific for human cMyC in (2 µg/mL TBS, pH 7.4) per well at RT. After blocking non-specific binding sites with blocking buffer (Roche) for 2 h, wells were washed three times with NaCl (0.154 M) containing Tween 20 (0.05 %). Hybridoma supernatants (50 µL) were then added to each well for 2 h. After extensive washing with NaCl-Tween, bound antibodies were detected by incubating the plates with a goat anti-mouse IgG, Fc-specific and conjugated to HRP, (Dianova, Hamburg, Germany) at a dilution of 1:10,000. Plates were read 10–15 min after adding the HRP substrate ABTS (Roche).

### Epitope mapping

The complete sequence of cMyC was synthesized as a library of 111 peptide spots (15mer) onto a cellulose membrane (Jerini, Berlin, Germany). The amino acid sequences of the peptides of neighboring spots were overlapping to ensure the continuous presentation of the complete cMyC sequence on the membrane. After washing the membrane once with methanol for 10 min and three times with TBS buffer and a casein-based blocking buffer (Cambridge Research Biochemicals, Cambridge, UK), it was incubated with the primary antibody (1 µg/mL) for 3 h at room temperature. The membrane was then washed three times with Tween-TBS buffer before the HRP-conjugated secondary antibody is added at 1 µg/mL for 2 h at room temperature. The chemiluminescent light detection of the recognized spots was performed on film after washing the membrane again three times in Tween-TBS buffer. Regeneration of the membrane for continued mapping was achieved with regeneration buffer (2 % SDS, 100 mM 2-mercaptoethanol, 50 mM Tris–HCl, pH 6.7).

### Ranking mouse monoclonal antibodies by surface plasmon resonance

To assess candidate anti-C0C2 monoclonal antibodies for their potential suitability, we utilized surface plasmon resonance (SPR) to rank the antibodies in terms of their kinetic parameters and establish whether a potential binding pair existed among the panel. The SPR experiments were performed using a Biacore 3000 or Biacore T200 (GE Healthcare) equipped with a research-grade s-series CM5 sensor chip. For kinetic experiments, the surfaces of two flow cells was activated for 5 min with a 1:1 mixture of 0.1 M NHS (*N*-hydroxysuccinimide) and 0.1 M EDC (3-(*N*,*N*-dimethylamino) propyl-*N*-ethylcarbodiimide) at a flow rate of 5 μL/min with HBS-EP (10 mM HEPES, 150 mM NaCl, 3.4 mM EDTA, 0.005 % Tween 20, pH 7.4) as running buffer. Goat anti-mouse IgG Fc-specific antibody (Sigma) was injected at a conc. of 30 µg/mL in 10 mM Na-acetate pH 5 and immobilized at a density of 3600 response units (RU) on flow cell 2 (Fc 2), whereas flow cell 1 was left blank to serve as a reference cell. Both flow cells were blocked with a 5 min injection of 1 M ethanolamine, pH 8.5, and the unbound material was removed with a 1 min injection of Gylcine-HCl, pH 2.5. To collect kinetic binding data, monoclonal antibodies in HBS-EP were firstly injected into the goat anti-mouse IgG-immobilized Fc 2, at 10 µL/min for 60 s to capture 100 RU of antibody, and allowed to stabilize for 600 s. C0C2 peptide in HBS-EP was then injected over the two flow cells at non-sequential concentrations of 200,100, 50 (×2), 25, 12.5 and 6.25 (×2) nM at a flow rate of 30 μL/min for 120 s at a temperature of 25 °C. The complex was allowed to dissociate for 800 s. The surfaces were regenerated with two 10 s injection of 25 mM NaOH, and signals obtained on the control surface and with three blank buffer injections were subtracted from the signal responses (double-reference). Data were collected at a rate of 1 Hz, and fitting performed using a simple 1:1 interaction model on the global data analysis option available within Biacore Biaevaluation software.

For competition studies, C0C2 peptide was directly conjugated to Biacore CM5 sensor chips (GE Biosciences) using the amine-coupling process described above. Subsequently, candidate antibodies (2 µg/mL) were injected at 10 µL/min in HBS-EP, and the change in RU was recorded. Successive injections of antibody continued until the sensorgram signal did not increase significantly and all available binding sites were deemed saturated with antibody. The competing antibody was then injected and the change in sensorgram signal was recorded until subsequent injections achieved saturation. The surface of the chip was then regenerated using 25 mM NaOH and the injection process was repeated, but with the antibody injection sequence reversed. The binding signal of each antibody in the presence of the competitor antibody was expressed as a percentage of the signal when the antibody was bound alone. A reduction in the binding signal >50 % in the presence of the second antibody was taken to indicate that the antibodies recognized overlapping epitopes.

### Quantitative immunoassay for cMyC

For the bioassay, a traditional “sandwich” format was adopted for use on an electrochemiluminescence (ECL) SECTOR imager 2400 instrument (MesoScale Discovery), by utilizing capture and detection monoclonal antibodies that recognized discrete epitopes on the C0C2 peptide. The capture antibody (Clone 3H8/30 µL/1 µg/mL) was coated onto 96-well SECTOR^®^ plates (MesoScale Discovery) in 10 mM Tris pH 9.6 overnight at 4 °C, washed three times with PBS/0.05 % Tween, and blocked with 1 %BSA/PBS for 1 hour at room temperature on a platform shaker. Serum samples (30 µL/well) were diluted 1 in 2 with Diluent 7 (MesoScale Discovery) and were added to the plate along with recombinant C0C2 standards also diluted with Diluent 7, before incubation for 1 h at room temperature. Afterwards, the plates were washed three times with PBS/0.05 % Tween to reduce non-specific binding. Detection antibody (Clone 1A4/30 µL/PBS pH 7.4), which had been conjugated to ruthenium (MesoScale Discovery) according to the manufacturer’s instructions, was then added to the wells and incubated for 2 h at room temperature with shaking at 300 rpm. Finally, the plates were washed three times with PBS/0.05 % Tween, and 150 µL 1× read buffer was added to the wells prior to ECL analysis on the SECTOR imager 2400. The standard curve generated was then used to quantify the My-C concentration present in serum samples and expressed as ng/L. The Limit of Detection (LOD) was calculated as 2.5 × standard deviation (SD) above the background signal and the Lower Limit of Quantification (LLoQ) as the lowest measured concentration above the LOD with a coefficient of variance <10 %.

### Statistics

We opted to measure time to peak concentration as our primary endpoint, since we envisaged our immunoblot and ELISA detection methods would lack the analytic sensitivity to compare time of first appearance (concentration exceeding limit of detection) of cMyC to that of Troponin. Since the timing of the appearance of cMyC in relation to cTn was unknown (this was the purpose of our study), a formal power calculation was not possible. We opted for 2-h collection intervals in our STEMI study, since this represents a time difference between peak cMyC and peak cTnT that would be clinically meaningful. Based on a non-parametric best case scenario if the cMyC peak were to precede or succeed the cTnT peak in each of the 20 patients, the likelihood of this arising by chance would be <0.00001. We therefore elected to study ≥20 patients with STEMI, a population size that was preserved for the subsequent cohorts. In this exploratory study, our secondary endpoints were rate of rise and fall of biomarker concentrations and absolute magnitude of biomarker concentration. The manner in which these comparisons were to be made was not prespecified. The time of peak concentration was not corrected for missing data points or for truncation of the dataset, if this value occurred in the last sample collected.

Whilst our ELISA lacked the sensitivity to measure cMyC before cardiac injury, within 15–30 min of injury, the concentration exceeded the limit of quantification defined by the concentration returning a 10 % coefficient of variation with the calibration standards. Thus, we were able to examine the rate of rise of cMyC versus cTnT in the first few hours after myocardial injury.

To correlate cMyC with cTnT, we compared the peak concentration of each analyte occurring within an individual patient undergoing either TASH or CABG using the Pearson correlation coefficient.

Results are presented as mean ± SEM. Comparison of time to peak was carried out using a paired, 2-tailed Student *t* test after testing for normality using a Kolmogorov–Smirnov test. The rates of accumulation and disappearance of biomarkers were compared using summary statistics obtained by linear (for accumulation) and monoexponential (for disappearance) fitting of serial values obtained from individual patients. All analyses were undertaken using GraphPad Prism 5 (GraphPad Software).

## Results

### cMyC release following ischemia in ex vivo and in vivo animal models

**Fig. 1:**
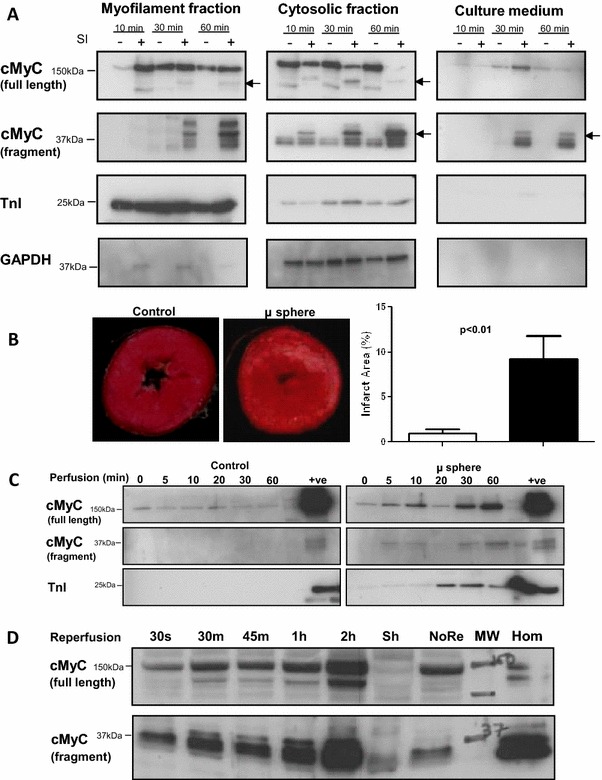
cMyC release following ischemia in ex vivo and in vivo animal models. **a** Redistribution and fragmentation of cMyC in cultured ARVMs subjected to simulated ischemia (SI). Cells and culture media were harvested after 10, 30 or 60 min of SI (+). Control cells and media were harvested at corresponding time points following equivalent media changes with non-ischemic buffer (−). SI causes increased fragmentation of cMyC with the dominant fragments at 40 kDa appearing in the cytosolic fraction and culture medium. The partitioning of cTnI differs from cMyC with immunoreactivity predominating in the myofilament fraction with later accumulation in the cytosolic fraction and no appreciable immunoreactivity, compared to cMyC, in the culture medium. **b** Myocardial infarct size assessment in Langendorff-perfused mouse hearts with and without the addition of microspheres to reduce coronary perfusate flow by 50 %. Right panels are representative Triphenyltetrazolium-stained short axis slices of hearts, taken at mid-ventricular level, 60 min after infusion of microspheres (μ sphere) or after time-matched buffer perfusion (Control). The microspheres caused patchy infarction of approximately 10 % of the left ventricular myocardial volume (see left pane, *n* = 6 for each group, *P* < 0.01). **c** Analysis of cMyC and cTnI release in pooled coronary perfusate from experiments depicted in (**b**). An increase in full-length and 40 kDa fragment of cMyC occurs during the course of perfusion after microsphere embolization. The time course of cTnI release is similar. There is a background level of cMyC detectable during control perfusion that most likely reflects on-going damage during perfusion. The +ve control was murine heart homogenate. **d** Appearance of cMyC in mouse serum following ligation of the anterior descending coronary artery. Mice were euthanized and exsanguinated at varied time points after 30 min of regional ischemia, as indicated. NoRe depicts no perfusion (150 min coronary ligation) and Sh depicts a sham procedure of 150 min of continued anesthesia without placement of a coronary ligature. Hom is heart homogenate used as a positive control. MW was reserved for molecular weight markers. The cTnI signal was not interpretable due to non-specific bands

Adult rat ventricular myocytes (ARVMs) were subjected to simulated ischemia (SI) before harvest and fractionation (Fig. 1a). The results demonstrate that the relative partitioning between soluble and insoluble fractions of cMyC and cTnI differ, with more cMyC in the soluble (cytosolic) fraction. Furthermore, SI causes fragmentation of cMyC with a dominant fragment appearing at 40 kDa (see arrows Fig. 1a). In contrast to cMyC, no cTnI protein was detected in the culture medium despite similar sensitivity of the anti-cMyC and anti-cTnI antibody conditions (see Figure 1 of supplemental data). To induce patchy myocardial infarction, akin to non-ST-elevation myocardial infarction (NSTEMI), we added sufficient microspheres to reduce coronary flow by 50 % which, after 60 min, resulted in approximately 10 % of the left ventricular myocardial volume undergoing infarction (see Fig. 1b). Figure 1c demonstrates that in the absence of microspheres, there is some elution of full-length cMyC, but no detectable cTnI, in keeping with minimal infarction. Following microspheres, there is an increase in both intact and fragmented cMyC and also in cTnI. Since perfusate is not recirculated, the results demonstrate on-going release of sarcomeric proteins. However, the temporal pattern of cMyC release is broadly similar to cTnI. Since the buffer is of low viscosity, the release profiles of cMyC and cTnI may not reflect those in vivo.

Following 30 min of left anterior descending (LAD) coronary artery ligation of the in situ heart, mice were euthanized and serum collected at several time points (see Fig. 1d). No cMyC release was detectable in sham (Sh)-operated controls. Whilst still easily detectable, cMyC release was diminished in the absence of reperfusion (NoRe). In common with ARVMs subjected to SI, additional degradation products are also visible containing the N-terminal C0C2 domains of cMyC. Unfortunately, it was not possible to reliably detect cTnI on immunoblots because of interference from mouse serum proteins. Taken together, these results confirm that cMyC is released from rodent myocardium at least as quickly as cTn and can be detected in the peripheral blood.

### Troponin T and cMyC release in patients with STEMI

**Fig. 2:**
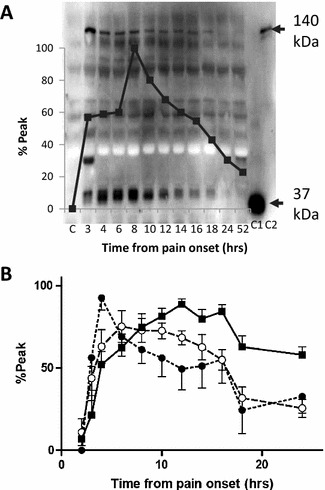
cMyC and cTnT accumulation during ST elevation myocardial infarction. **a** cMyC immunoblot of serum collected from an individual patient with the corresponding laboratory-determined cTnT concentrations appearing as an overlay. The controls are C1, recombinant C0C2 domain of cMyC; C2, fresh mouse heart homogenate and C, control human serum. Soon after chest pain onset cMyC appears in the serum predominantly as the full-length protein (~140 kDa) which then diminishes as a shorter fragment accumulates (~40 kDa). These fragments seem to reach maximum abundance in advance of cTnT. **b** Relative timings of cMyC, measured by densitometry of full-length protein (*closed circles*) and dominant fragment (*open circles*), compared to cTnT (*filled squares*) measured in our clinical laboratory. The immunoblot detection technique is relatively crude and insensitive (see text for details), so band densities are expressed as % of the maximum density achieved at a single time point on the individual patient’s immunoblot (see **a** for example). Complete values for cTnT were obtained after immunoblot analyses and normalized similarly. The peak concentrations of full-length (7.9 ± 4.4 h) and fragment (9.3 ± 3.1 h) forms of cMyC occurred earlier than those of Troponin (11.8 ± 3.4 h, *P* < 0.007 and *P* < 0.001, respectively). The figure comprises analyses from 18 patients for cMyC fragment and cTnT (*n* = 18). In two patients, there was a serum sample missing at the peak of troponin concentration and in two patients no clear cMyC immunoreactivity could be discerned. Full-length cMyC could only be detected in the serum of 9 of the 18 patients in whom a fragment was visible

We compared release of cMyC to cTnT by examining the time to peak concentration in serum samples from 20 STEMI patients presenting early after chest pain onset (patient characteristics are shown in Table 1 of the online supplement) with a cTnT concentration 12 h after admission >2000 ng/L. Venous blood was collected on admission and then every 2 h until 18 h after symptom onset and then once again at 24 h. The main purpose was to visualize any cMyC-circulating fragments. Since we were unable to reliably detect cMyC immunoreactivity if cTnT peak concentration was less than 2000 ng/L, we estimate the difference between the lower limit of detection of cMyC by immunoblot and cTnT by commercial electrochemiluminescent high-sensitivity immunoassay (Roche Elecsys E170) to be at least two orders of magnitude. This difference precluded a comparison based on time at which the biomarker was first detected. For this reason, our a priori intent was to compare time to peak concentration of cMyC and cTnT. Figure 2a shows the pattern of cMyC immunoreactivity in a patient presenting 180 min after chest pain onset. In this particular patient, the peak concentration of cMyC occurred much earlier than that of cTnT, an advantage particularly apparent for the full-length protein (approx. 140 kDa, see upper arrow). We performed an identical analysis in patients presenting early with suspected STEMI. Of the 20 STEMI patients’ serum we analyzed, there were two in which there were no clear cMyC bands despite a substantial cTnT rise. Thus, 18 patients subsequently proved to have sufficient myocardial infarction to allow detection of at least the 40 kDa fragment of cMyC (see lower band on Fig. 2a). Figure 2b shows the relative concentration profiles from time of presentation after chest pain onset (2–14 h, mean 3.3 h) to 24 h. This information was collected in advance of the laboratory determination of cTnT in the research samples. There is fairly rapid accumulation of the full-length cMyC protein (filled circles) with a mean time to peak of 7.9 ± 4.4 h that precedes the more easily detected 40 kDa fragment (open circles) with a mean time to peak of 9.3 ± 3.1 h. Both compare favorably with cTnT (closed squares) (time to peak of 11.8 ± 3.4 h, *P* > 0.007 and *P* > 0.001, respectively). In every patient’s profile, the time of peak concentration of full-length cMyC preceded that of cTnT (9/9) (*P* > 0.007) whilst for the 40 kDa fragment it coincided or preceded it in 16/16 (*P* > 0.001). Although our immunoblotting technique for cMyC detection was relatively crude and insensitive, it suggested that the cMyC release kinetics compare favorably with cTnT, prompting investment in a more sensitive and quantitative assay.

### The creation of an immunoassay based on novel monoclonal antibodies

**Fig. 3:**
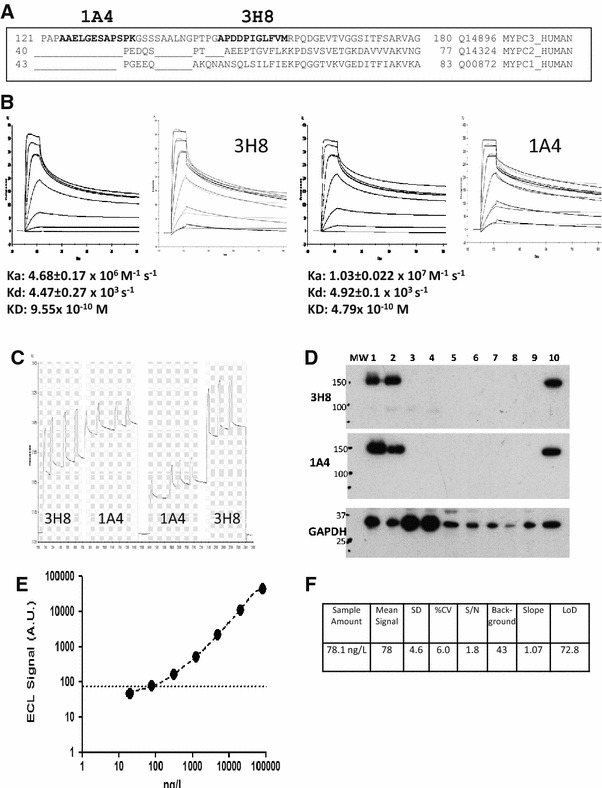
Development of a quantitative bioassay for human cMyC in serum. **a** Sequence alignment of cMyC with skeletal myosin binding protein C isoforms. The sequences recognized by monoclonal anti-cMyC antibodies 1A4 and 3H8 are shown in *bold*. **b** SPR kinetic sensorgrams demonstrating the kinetic parameters of clone 3H8 (*left*) and 1A4 (*right*). **c** Epitope competition sensorgram of 1A4 and 3H8 binding to a C0C2-conjugated CM5 chip. **d** Immunoblot of rat and human tissue demonstrating specificity of 3H8 and 1A4 monoclonal antibodies. GAPDH was used as a loading control. Samples *1*–*9* are various rat tissue (*1* ventricle, *2* atria, *3* rectus abdominus, *4* soleus, *5* spleen, *6* kidney, *7* aorta, *8* liver, *9* brain) and *10* is human ventricle. e Representative C0C2 standard curve from cMyC ElectroChemiLuminescent assay indicating the limit of detection (*dashed line*). f The performance characteristics of the assay for MyC, with a lower limit of quantification at a 10 % coefficient of variation, of approximately 80 ng/L

We chose to develop a classic “sandwich” immunoassay format, comprising a single monoclonal capture and a single monoclonal detection antibody, both directed against unique epitopes on the cardiac-specific N-terminal domain of cMyC (see Fig. 3a). An array of 15mer peptides corresponding to overlapping sections of this C0C2 domain was used to map epitope specificity. From 74 viable hybridomas, clones 1A4 and 3H8 were chosen on biophysical characteristics (see below) and mapped to sequences A_125_AELGESAPSPK and A_149_PDDPIGLFVM, respectively (see emboldened sequence in Fig. 3a). The 3H8 (capture), and 1A4 (detection) antibodies exhibited strong binding affinity to C0C2 of 9.6 × 10^−10^ and 4.8 × 10^−10^ M by surface plasmon resonance (for sensorgram see Fig. 3b). The epitope competition sensorgram (Fig. 3c) shows that 3H8 and 1A4 can bind to C0C2 independently, despite the proximity of their epitopes demonstrated by peptide array (see Fig. 3a). Monoclonal 3H8 bound at 80 % of its maximum binding value in the presence of saturating amounts of 1A4, whereas 1A4 bound at 68 % of its maximum value after saturating injections of 3H8. These high non-competing affinities were accompanied by specificity for cMyC over other isoforms based on immunoblots of rat and human tissue (Fig. 3d).

We next incorporated our selected mAbs into a classic “sandwich” immunoassay using electrochemiluminesence (ECL) as it forms the basis of the Roche Elecsys hs-cTnT system. A typical ECL assay standard curve is shown (see Fig. 3e), with the current limit of quantitative detection (80 ng/L) indicated by the dashed line. We, therefore, used this quantitative assay to further define the kinetics of cMyC in human serum.

### The accumulation of cMyC versus cTnT in the serum after timed myocardial injury

**Fig. 4:**
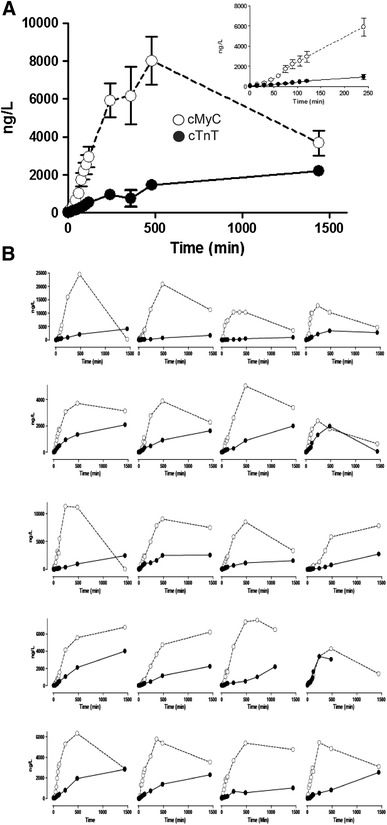
The accumulation of cMyC compared to cTnT after myocardial injury caused by intracoronary ethanol. Venous blood was collected frequently over the first 4 and up to 24 h after TASH using ethanol infused selectively into a perforating branch coronary artery. **a** Summary data of absolute quantification of cMyC (*open symbols*) versus cTnT (*closed symbols*) over time following TASH (*n* = 20). *Inset* *figure* is a zoom of the first 240 min. Over this time interval, cMyC accumulates in the serum approximately 6 times faster than TnT (slope 25.8 ± 1.9 vs 4.0 ± 0.4 ng/L/min, *P* < 0.0001). **b** Shows the concentration versus time relationship for cMyC and cTnT for each of the 20 individual patients summarized on (**a**)

The disadvantages of the STEMI cohort are that serum sampled early after coronary occlusion is not available and that the timing of coronary artery occlusion is imprecise. A further difficulty was sample depletion due to the high serum volume required to run the immunoblot-based assay. Furthermore, all patients underwent primary PCI, potentially confounding biomarker release kinetics. For all these reasons, we used serum collected prospectively from 20 patients undergoing elective therapeutic ablation of septal hypertrophy (TASH) by selective intracoronary injection of ethanol to relieve left ventricular outflow tract obstruction caused by hypertrophic cardiomyopathy. An overlapping patient cohort has been previously described [22]. Venous serum samples were collected before and at 15, 30, 45, 60, 75, 90 and 105 min and at 2, 4, and 24 h after TASH. Figure 4a shows the concentration of cMyC (open symbols), determined using the assay described above, and of high-sensitivity cTnT (closed symbols), determined using an Elecsys Analyser (2010 or E170, Roche Diagnostics). Of the pre-procedural concentrations, 2/20 for cMyC, and 11/20 for cTnT, were above the limit of reliable quantitation (defined as concentration returning ≤10 % coefficient of variation during standard calibration on day of assay for cMyC and 14 ng/L for cTnT). Nonetheless, there was a significant increase in concentration of both biomarkers within the first 15 min (preprocedure vs 15 min, cMyC 41.7 ± 16.7 vs 181.5 ± 41.4 ng/L; *P* = 0.0008 and hscTnT 22.5 ± 5.5 vs 53.7 ± 17.3 ng/L; *P* = 0.03). Overall, the rate of rise for cMyC was faster than that of TnT and in aggregate, the serum concentrations had begun to fall, whilst cTnT was still rising. Simple linear regression was used to fit the untransformed concentration versus time relationship over the first 240 min for individual patients (see inset of Fig. 4a for aggregate data). This simple model provided a reasonable fit (cMyC *R* ^2^ = 0.49, Sy.x = 1639, *P* < 0.0001 and TnT *R* ^2^ = 0.31, Sy.x = 378, *P* < 0.0001) and demonstrates that cMyC accumulates in the serum approximately 6 times faster than cTnT (slope 25.8 ± 1.9 vs 4.0 ± 0.4 ng/L/min, *P* < 0.0001).

The aggregate data do not provide an adequate overview of the inter-patient variability of cMyC and cTnT accumulation in serum. Figure 4b displays this relationship for each individual patient. Whilst there is considerable variation, generally cMyC reaches its peak concentration at or before 4 h, whilst cTnT is still rising at 24 h. The slow rise of TnT in this model has been noted previously [22].

### The clearance of cMyC v cTnT in the serum after timed myocardial injury

**Fig. 5:**
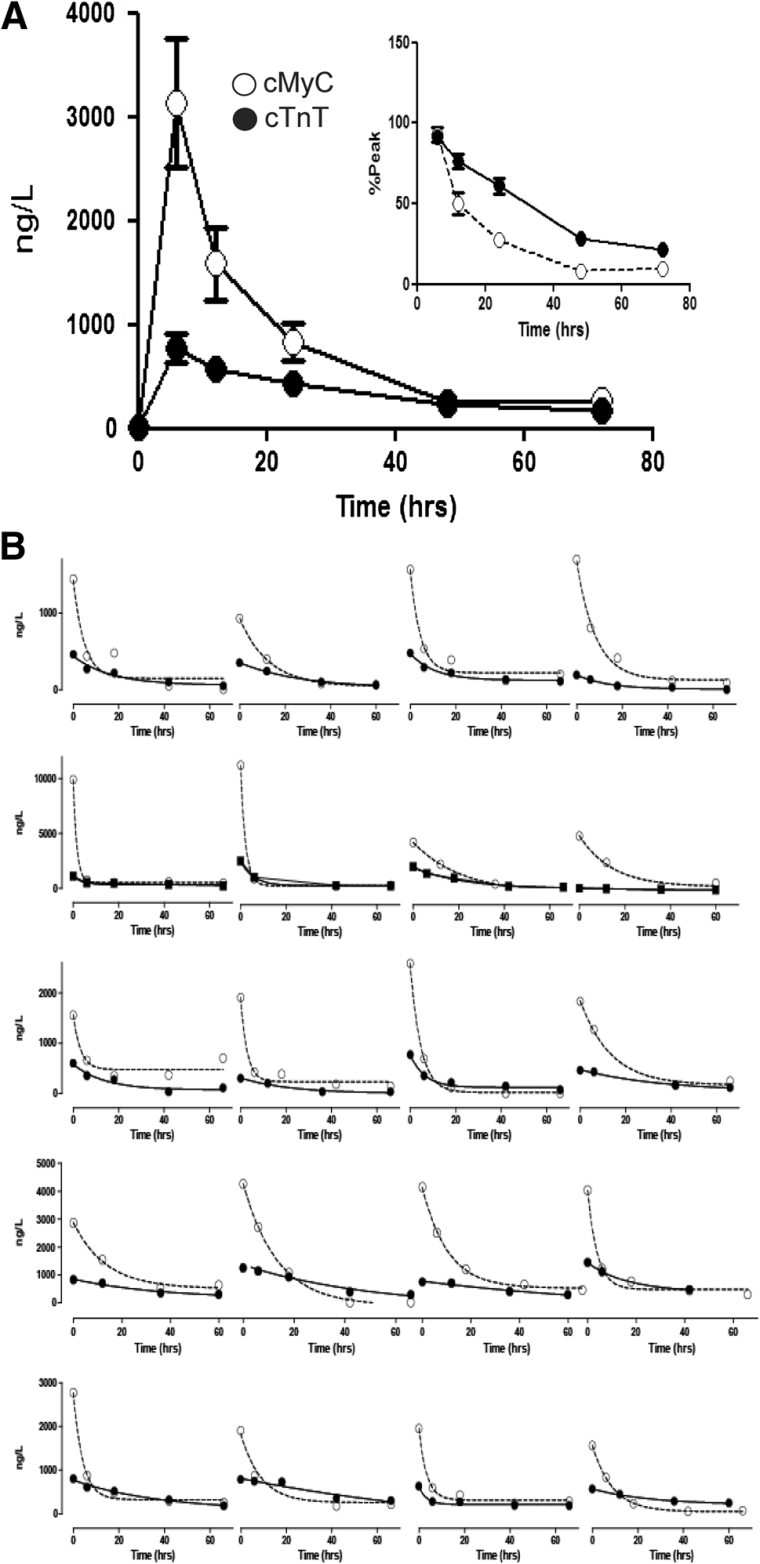
The accumulation of cMyC (*open symbols*) compared to cTnT (*closed symbols*) after myocardial injury caused by surgical revascularization. Venous blood was collected over 3 days following CABG. **a** Summary data of absolute quantification of cMyC versus cTnT over time following CABG (*n* = 20). *Inset* *figure* is a zoom of the last five time points expressed as a % of peak concentration achieved in each patient. This normalization was used to remove the visual bias caused by the greater absolute concentration of cMyC. **b** Shows the concentration versus time relationship for cMyC and cTnT for each of the 20 individual patients summarized on (**a**). The monoexponential used for curve-fitting to determine clearance half-times is drawn as a *continuous line*. The decay half-time for cMyC is considerably shorter than for cTnT (5.5 ± 0.8 vs 22 ± 5 h, *P* < 0.0001)

The duration of admission for STEMI and TASH is too short to get an adequate profile of clearance. To examine this we used serum collected during an investigation of the effects of ciclosporin on cardiac protection during coronary artery bypass surgery where myocardial injury was assessed using serum biomarkers collected before, and 6, 12, 24, 48 and 72 h after surgery. The biomarker release profile differs markedly from that during TASH (compare Figs. 4a to 5a) with both markers achieving their peak concentration at 6 h, with cMyC (open symbols) falling substantially at 12 h. This probably reflects myocardial washout by brisk reperfusion on release of the aortic cross clamp. Figure 5b shows comparative rates of clearance of cMyC and hscTnT (closed symbols) from the serum for individual patients with corresponding monoexponential curves of best fit. Based on these time constants, the decay half-time for cMyC is considerably shorter than for cTnT (5.5 ± 0.8 vs 22 ± 5 h, *P* < 0.0001).

**Fig. 6:**
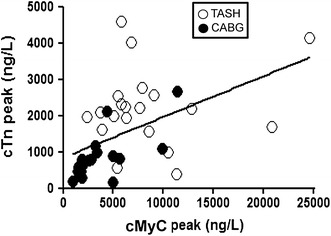
The relationship between cMyC and cTnT. The maximum serum concentration achieved for cMyC and cTnT in the series of blood draws for patients undergoing TASH (*open symbols*) or CABG (*closed symbols*) was compared by linear regression. *R* ^2^ = 0.30, *P* < 0.0001, [cTnT] = 0.11x[cMyC] + 845

To gain some idea of the comparative concentrations of cMyC and cTnT, in Fig. 6, we compared the peak concentration achieved for each biomarker in the CABG (closed symbols) and TASH (open symbols) cohorts. There is a reasonable correlation between peak cTnT and peak cMyC (see Fig. 6, *R* ^2^ = 0.3, *P* < 0.0001) which compares favorably with the best correlation between four high sensitivity cTn assays applied to a healthy population (*R* ^2^ = 0.08) [2]. This relationship is closest for the CABG cohort, since it is likely the peak cTnT concentration was not recorded in the TASH cohort (occurred after 24 h). On average, the peak cMyC concentration is fivefold greater than the peak cTn concentration, despite cMyC calibration with a fragment rather than with the full-length protein.

## Discussion

Our exploratory study is the first to quantify the timing and forms of cMyC released following myocardial injury in animal models and in patients with myocardial infarction. We found that cMyC accumulates and disappears more rapidly than cTnT. We hypothesize that these characteristics suggest cMyC could provide supplementary information that may impact on the triage and subsequent management of patients presenting with chest pain.

This was an experimental study to further characterize a novel, cardiac-specific biomarker of AMI and to determine if it provides any potential additional information to cTnT. Our assessment was limited to very well-defined patient groups in whom there is documented and substantial myocardial injury. These groups did not include patients with NSTE-ACS, the patient group of most relevance to our putative biomarker. Our current assay is not suitable for a “real world” evaluation against, or in addition to, cTns since it lacks comparable analytic performance. To determine if cMyC offers any diagnostic advantage, it will be necessary to generate a more sensitive assay capable of measuring cMyC concentrations in healthy individuals, without myocardial injury, to define the 99th percentile cutoff with which to diagnose AMI [31]. If we were to attempt this with our current assay, we would return analytic, rather than biologic, variation. Furthermore, we have not undertaken a quantitative comparison between cMyC and cTnI. Nonetheless, it is tempting to speculate how our findings could potentially translate to clinical practice.

Over the last decade, the commercial cTn assays have achieved astounding sensitivity but many patients without acute myocardial injury have cTn concentrations above the 99th percentile defined by a healthy reference population [20]. Consequently, if this concentration is used as the diagnostic cutoff, the positive predictive value can be as low as 50 %, even in relatively high-risk populations attending emergency units at academic medical centers [13, 26, 27]. Accordingly, two serum estimations maybe needed to determine the magnitude of change in concentration. Unfortunately it is still not clear whether this strategy will dramatically increase specificity, because of the slow kinetics of cTn release and the known biological variation in cTn concentration in patients with stable heart disease [18]. Our study suggests cMyC may be able to overcome this disadvantage by rising more rapidly than the cTns. This characteristic maybe further enhanced by the apparent more rapid clearance of cMyC preventing the integration of the subclinical events that presumably contribute to cTn concentrations in ambulatory patients being elevated above the 99th percentile. However, these theoretical advantages can only be tested once the sensitivity of MyC assay is improved.

The findings we report here are in keeping with others reported recently by Gonvindan et al. [11] and Kuster et al. [21] that also suggest cMyC is released more rapidly than cTnT. However, in contrast to the current study, these previous studies were unable to perform a quantitative comparison of cMyC against cTnT since the diagnostic performance of the assays employed for both analytes was poor (lower limit of quantification with 20 % coefficient of variation >640 ng/L). Consequently, the concentrations of cMyC in healthy controls (22.3 ± 2.4 µg/L) were approximately 1000-fold higher than those we report with a dynamic range of only five during AMI (105.1 ± 8.8 µg/L) [11]. Similarly a significant rise in cTnT was only detected 6 h after alcohol septal ablation [21] in contrast to 15 min in the current, and a previous, study [22], despite samples originating from the same source.

In the absence of a normal cutoff concentration for cMyC defined by the 99th percentile of a healthy population, much of our discussion is necessarily speculative. We estimate that our assay for cMyC needs to be approximately fivefold more sensitive to measure serum concentrations in a substantial proportion of healthy individuals. This estimation is based on the cMyC concentration that returns a 10 % coefficient of variation (approximately 100 ng/L) combined with abundance in serum that is approximately fivefold higher than that of cTnT.

In addition to the established and pure markers of myocardial necrosis, cTnT and cTnI, a range of other biomarkers have been proposed to aid triage in suspected NSTE-ACS. These include C-terminal preprovasopressin (copeptin), interleukin 33 (ST2), growth differentiation factor 15 (GDF15), heart-specific fatty acid-binding protein (HFAB), ischaemia-modified albumin (IMA), myoglobin and creatine kinase MB (CK.MB). Since these markers are not cardiac-restricted, they lack the specificity of the cTns for the diagnosis of AMI in suspected NSTE-ACS; however, they may possess higher sensitivity. However, this potential advantage is negated by their poor specificity, such that generally their diagnostic performance is lower than that of the cTns [23, 29]. In contrast to these new markers, the expression of cMyC is cardiac-restricted and its release should be specific for circumstances associated with cardiac injury. Thus, cMyC may have the sensitivity advantages of the non-cTn biomarkers, but without their limitations in specificity.

## Conclusions

This exploratory translational study suggests cMyC is a novel biomarker of myocardial injury with kinetics that differs significantly from cTnT. A sensitive cMyC assay is a prerequisite for further validation and comparison to cTns in the relevant patient population with suspected NSTE-ACS.

## Electronic supplementary material

Below is the link to the electronic supplementary material.

Supplementary material 1 (DOCX 697 kb)

## Abbreviations

ACS: Acute coronary syndrome
AMI: Acute myocardial infarction
ARVMs: Adult rat ventricular myocytes
CABG: Coronary artery bypass graft surgery
cMyC: Cardiac myosin-binding protein C
cTn: Cardiac troponin
ECL: Electrochemiluminesence
Ig: Immunoglobulin
LAD: Left anterior descending coronary artery
NSTE-ACS: Non-ST elevation acute coronary syndromes
NSTEMI: Non-ST elevation myocardial infarction
SI: Simulated ischemia
Sh: Sham operated
SPR: Surface plasmon resonance
STEMI: ST-elevation myocardial infarction
TASH: Transcoronary therapeutic ablation of septal hypertrophy

## References

[1] Aldous SJ, Richards AM, Cullen L, Than MP (2011). Early dynamic change in high-sensitivity cardiac troponin T in the investigation of acute myocardial infarction. Clin Chem.

[2] Apple FS, Ler R, Murakami MM (2012). Determination of 19 cardiac troponin I and T assay 99th percentile values from a common presumably healthy population. Clin Chem.

[3] Aye TT, Scholten A, Taouatas N, Varro A, Van Veen TA, Vos MA, Heck AJ (2010). Proteome-wide protein concentrations in the human heart. Mol BioSyst.

[4] Baker JO, Devaraj R, Reinhold J, Kanaganayagam G, Sadayappan S, Gautel M, Redwood S, Marber M (2010). Cardiac myosin-binding protein C as a potential new serum biomarker of myocardial infarction. Circulation.

[5] Bellahcene M, Jacquet S, Cao XB, Tanno M, Haworth RS, Layland J, Kabir AM, Gaestel M, Davis RJ, Flavell RA, Shah AM, Avkiran M, Marber MS (2006). Activation of p38 mitogen-activated protein kinase contributes to the early cardiodepressant action of tumor necrosis factor. J Am Coll Cardiol.

[6] Clark JE, Kottam A, Motterlini R, Marber MS (2009). Measuring left ventricular function in the normal, infarcted and CORM-3-preconditioned mouse heart using complex admittance-derived pressure volume loops. J Pharmacol Tox Methods.

[7] Cooper A, Timmis A, Skinner J (2010). Assessment of recent onset chest pain or discomfort of suspected cardiac origin: summary of NICE guidance. BMJ.

[8] de Lemos JA, Morrow DA, deFilippi CR (2011). Highly sensitive troponin assays and the cardiology community: a love/hate relationship?. Clin Chem.

[9] De Nicola G, Martin E, Chaikuad A, Bassi R, Clark J, Martino L, Verma S, Sicard P, Tata R, Atkinson R, Knapp S, Conte M, Marber M (2013). Mechanism and consequence of the autoactivation of p38? mitogen-activated protein kinase promoted by TAB 1. Nat Struct Mol Biol.

[10] Gerszten RE, Carr SA, Sabatine M (2010). Integration of proteomic-based tools for improved biomarkers of myocardial injury. Clin Chem.

[11] Govindan S, Kuster DW, Lin B, Kahn DJ, Jeske WP, Walenga JM, Leya F, Hoppensteadt D, Fareed J, Sadayappan S (2013). Increase in cardiac myosin binding protein-C plasma levels is a sensitive and cardiac-specific biomarker of myocardial infarction. Am J Cardiovasc Dis.

[12] Hammarsten O, Fu ML, Sigurjonsdottir R, Petzold M, Said L, Landin-Wilhelmsen K, Widgren B, Larsson M, Johanson P (2012). Troponin T percentiles from a random population sample, emergency room patients and patients with myocardial infarction. Clin Chem.

[13] Hoeller R, Rubini Gimenez M, Reichlin T, Twerenbold R, Zellweger C, Moehring B, Wildi K, Freese M, Stelzig C, Hartmann B, Stoll M, Mosimann T, Reiter M, Haaf P, Mueller M, Meller B, Hochgruber T, Balmelli C, Sou SM, Murray K, Freidank H, Steuer S, Minners J, Osswald S, Mueller C (2013). Normal presenting levels of high-sensitivity troponin and myocardial infarction. Heart.

[14] Jacquet S, Nishino Y, Kumphune S, Sicard P, Clark JE, Kobayashi KS, Flavell RA, Eickhoff J, Cotten M, Marber MS (2008). The role of RIP2 in p38 MAPK activation in the stressed heart. J Biol Chem.

[15] Jacquet S, Yin X, Sicard P, Clark J, Kanaganayagam GS, Mayr M, Marber MS (2009). Identification of cardiac myosin-binding protein C as a candidate biomarker of myocardial infarction by proteomics analysis. Mol Cell Proteomics.

[16] Katus HA, Remppis A, Neumann FJ, Scheffold T, Diederich KW, Vinar G, Noe A, Matern G, Kuebler W (1991). Diagnostic efficiency of troponin T measurements in acute myocardial infarction. Circulation.

[17] Keller T, Zeller T, Peetz D, Tzikas S, Roth A, Czyz E, Bickel C, Baldus S, Warnholtz A, Frohlich M, Sinning CR, Eleftheriadis MS, Wild PS, Schnabel RB, Lubos E, Jachmann N, Genth-Zotz S, Post F, Nicaud V, Tiret L, Lackner KJ, Munzel TF, Blankenberg S (2009). Sensitive troponin I assay in early diagnosis of acute myocardial infarction. N Engl J Med.

[18] Klinkenberg LJ, van Dijk JW, Tan FE, van Loon LJ, van Dieijen-Visser MP, Meex SJ (2014). Circulating cardiac troponin T exhibits a diurnal rhythm. J Am Coll Cardiol.

[19] Kohler G, Milstein C (1975). Continuous cultures of fused cells secreting antibody of predefined specificity. Nature.

[20] Korley FK, Jaffe AS (2013). Preparing the United States for high-sensitivity cardiac troponin assays. J Am Coll Cardiol.

[21] Kuster DW, Cardenas-Ospina A, Miller L, Liebetrau C, Troidl C, Nef HM, Mollmann H, Hamm CW, Pieper KS, Mahaffey KW, Kleiman NS, Stuyvers BD, Marian AJ, Sadayappan S (2014). Release kinetics of circulating cardiac myosin binding protein-C following cardiac injury. Am J Physiol Heart Circ Physiol.

[22] Liebetrau C, Mollmann H, Nef H, Szardien S, Rixe J, Troidl C, Willmer M, Hoffmann J, Weber M, Rolf A, Hamm C (2012). Release kinetics of cardiac biomarkers in patients undergoing transcoronary ablation of septal hypertrophy. Clin Chem.

[23] Lipinski MJ, Escarcega RO, D’Ascenzo F, Magalhaes MA, Baker NC, Torguson R, Chen F, Epstein SE, Miro O, Llorens P, Giannitsis E, Lotze U, Lefebvre S, Sebbane M, Cristol JP, Chenevier-Gobeaux C, Meune C, Eggers KM, Charpentier S, Twerenbold R, Mueller C, Biondi-Zoccai G, Waksman R (2014). A systematic review and collaborative meta-analysis to determine the incremental value of copeptin for rapid rule-out of acute myocardial infarction. Am J Cardiol.

[24] Marber MS (2000). Ischemic preconditioning in isolated cells. Circ Res.

[25] Morrow DA (2009). Clinical application of sensitive troponin assays. N Engl J Med.

[26] Reichlin T, Hochholzer W, Bassetti S, Steuer S, Stelzig C, Hartwiger S, Biedert S, Schaub N, Buerge C, Potocki M, Noveanu M, Breidthardt T, Twerenbold R, Winkler K, Bingisser R, Mueller C (2009). Early diagnosis of myocardial infarction with sensitive cardiac troponin assays. N Engl J Med.

[27] Reichlin T, Irfan A, Twerenbold R, Reiter M, Hochholzer W, Burkhalter H, Bassetti S, Steuer S, Winkler K, Peter F, Meissner J, Haaf P, Potocki M, Drexler B, Osswald S, Mueller C (2011). Utility of absolute and relative changes in cardiac troponin concentrations in the early diagnosis of acute myocardial infarction. Circulation.

[28] Sadayappan S, de Tombe PP (2012). Cardiac myosin binding protein-C: redefining its structure and function. Biophys Rev.

[29] Schaub N, Reichlin T, Twerenbold R, Reiter M, Steuer S, Bassetti S, Stelzig C, Wolf C, Winkler K, Haaf P, Meissner J, Drexler B, Mueller C (2012). Growth differentiation factor-15 in the early diagnosis and risk stratification of patients with acute chest pain. Clin Chem.

[30] Snabaitis AK, D’Mello R, Dashnyam S, Avkiran M (2006). A novel role for protein phosphatase 2A in receptor-mediated regulation of the cardiac sarcolemmal Na+/H+ exchanger NHE1. J Biol Chem.

[31] Thygesen K, Alpert JS, Jaffe AS, Simoons ML, Chaitman BR, White HD, Katus HA, Lindahl B, Morrow DA, Clemmensen PM, Johanson P, Hod H, Underwood R, Bax JJ, Bonow RO, Pinto F, Gibbons RJ, Fox KA, Atar D, Newby LK, Galvani M, Hamm CW, Uretsky BF, Steg PG, Wijns W, Bassand JP, Menasche P, Ravkilde J, Ohman EM, Antman EM, Wallentin LC, Armstrong PW, Januzzi JL, Nieminen MS, Gheorghiade M, Filippatos G, Luepker RV, Fortmann SP, Rosamond WD, Levy D, Wood D, Smith SC, Hu D, Lopez-Sendon JL, Robertson RM, Weaver D, Tendera M, Bove AA, Parkhomenko AN, Vasilieva EJ, Mendis S (2012). Third universal definition of myocardial infarction. Circulation.

