# Supplementary material for: Cardiac myosin-binding protein C: a potential early biomarker of myocardial injury
Source: Basic Res Cardiol. 2015 Apr 3;110(3):23. doi: 10.1007/s00395-015-0478-5 (PMC4383815; doi:10.1007/s00395-015-0478-5)
Supplement: Supplementary file 1 — Supplementary material 1 (DOCX 697 kb) [file 395_2015_478_MOESM1_ESM.docx]

**Online Appendix for the following article:**

**TITLE**: **CARDIAC MYOSIN-BINDING PROTEIN C: A POTENTIAL EARLY BIOMARKER OF MYOCARDIAL INJURY**

**AUTHORS**: James O Baker^1§^ BSc MD, Raymond Tyther^1§^ PhD, Christoph Liebetrau^2^ MD, James Clark^1^ PhD, Tiffany Patterson^1^ MB.BS, Helge Möllmann^2^ MD, Holger Nef^2^ MD, Pierre Sicard^1^ PhD, Balrik Kailey^1^ BSc, Renuka Devaraj^1^ MSc, Simon R Redwood^1^ MD, Gudrun Kunst^3^ MD PhD, Ekkehard Weber^4^ PhD, Michael S Marber^1^ MB.BS, PhD

**SUPPLEMENTAL RESULTS
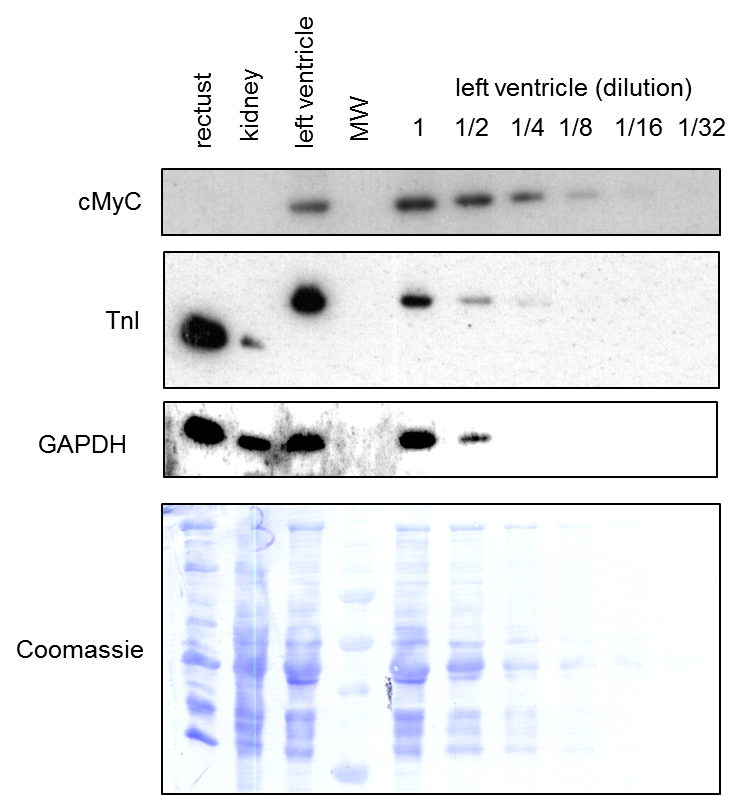
**

**Figure 1. Comparison between primary antibodies against cMyC and TnI.** Polyclonal antibodies against TnI and cMyC were used as described in the methods and figure 1 of the main manuscript. Above they are compared against serial dilution of the left ventricle sample that appears in lane 3 (left ventricle). The two antibodies have broadly similar sensitivity. The anti-TnI antibody recognises skeletal muscle (rectus=rectus abdominis muscle). The immunoreactivity in the kidney lane seen with the TnI antibody is likely sample spillover from the adjacent lane. The immunoblot against Glyceraldehyde 3-phosphate dehydrogenase (GAPDH) and the Coomasie Blue-stained gel are included to illustrate protein loading.

| **Cohort** | STEMI  (n=20) | TASH  (n=20) | CABG  (n=20) |
| --- | --- | --- | --- |
| **Male (%)** | 80 | 45 | 80 |
| **Age** | 65.0(1.7) | 61.9(2.6) | 64.6(2.2) |
| **Time to presentation (min)** | 195.3(41) | N/A | N/A |
| **Presentation to reperfusion (min)** | 61.3(4.3) | N/A | N/A |
| **Diabetes Mellitus(%)** | 25 | 20 | 25 |
| **Hypertension (%)** | 35 | 65 | 80 |
| **Dyslipidaemia (%)** | 35 | 55 | 65 |
| **Smokers (%)**  **Estimated GFR (mL/min)**  **Aortic cross clamp time (min)**  **Bypass time** | 25  N/A  N/A  N/A | 40  82.7(6.2)  N/A  N/A | 30  74(3.1)  52(3.8)  89(6.0) |

**Supplemental Table 1 Baseline demographics of study populations**

Data are mean (sem)
